# Assessment of pregnancy-associated glycoprotein profile in milk for early pregnancy diagnosis in goats

**DOI:** 10.5713/ajas.19.0399

**Published:** 2020-01-13

**Authors:** Shiva Pratap Singh, Ramachandran Natesan, Nandini Sharma, Anil Kumar Goel, Manoj Kumar Singh, Suresh Dinkar Kharche

**Affiliations:** 1 Animal Physiology and Reproduction Division, Indian Council of Agricultural Research-Central Institute for Research on Goats, Makhdoom, Farah, Mathura, U.P., 281122, India; 2 Animal Genetics and Breeding Division, Indian Council of Agricultural Research-Central Institute for Research on Goats, Makhdoom, Farah, Mathura, U.P., 281122, India

**Keywords:** Pregnancy-associated Glycoprotein, Milk, Goat, Pregnancy Diagnosis

## Abstract

**Objective:**

This study was conducted to assess the level of pregnancy-associated glycoprotein (PAG) in whole and skim milk samples, and its suitability for early pregnancy diagnosis in goats.

**Methods:**

A two-step sandwich enzyme-linked immunosorbent assay (ELISA) system for estimation of milk PAG was developed and validated, which employed caprine-PAG specific polyclonal antisera. Whole and skim milk samples (n = 210 each) from fifteen multiparous goats were collected on alternate days from d 10 to d 30, and thereafter weekly till d 51 post-mating. PAG levels in milk samples were estimated by ELISA and the pregnancies were confirmed at d40 post-mating by transrectal ultrasonography (TRUS).

**Results:**

The level of PAG in whole and skim milk samples of both pregnant and non-pregnant goats remained below the threshold values until d 24 after mating. Thereafter, PAG concentration in whole and skim milk increased steadily in pregnant goats, whereas it continued below the threshold in non-pregnant does. The PAG profiles in whole and skim milk of pregnant goats were almost similar and exhibited strong positive relationship (r = 0.891; p<0.001). Day 26 post-mating was identified as the first time-point for significantly (p<0.05) higher milk PAG concentration in pregnant goats than to non-pregnant goats. When compared to TRUS examination for pregnancy diagnosis, the accuracy and specificity of PAG ELISA using whole and skim milk samples were 94.5% and 95.4%; and 95.3% and 100%, respectively. The high values of area-under-curve (0.904 [whole milk] and 0.922 [skim milk]), demonstrate outstanding discrimination ability of the milk assays. Among the sampling dates chosen, d 37 post-mating was identified as the best suitable time point for collection of milk samples to detect pregnancy in goats.

**Conclusion:**

The PAG concentration in whole and skim milk of goats collected between days 26 and 51 post-breeding can be used for the accurate prediction of pregnancy and may be useful for assisting management decisions in goat flocks.

## INTRODUCTION

An early and accurate diagnosis of pregnancy after breeding improves pregnancy rate and reproductive efficiency of farm animals. In goats, diagnosis of pregnancy by transrectal ultrasonography (TRUS) method is possible from d 21 after breeding [1]. However, for scanning and interpretation of the results, specialized equipment and expertise are required. Further, its accuracy has not reached to 100% before d 30 post-breeding in most of the studies [2,3]. Instead, early pregnancy diagnosis can be done before d 30 of gestation using milk based assays in farm animals such as cattle [4,5] and sheep [6].

Pregnancy-associated glycoproteins (PAG), a large family of inactive aspartic proteinase, are solely secreted from mono and bi-nucleate cells of trophoectoderm [7]. Thus, as a more specific biomarker, PAG may provide an alternate possibility for early pregnancy diagnosis in goats. Use of plasma level of PAG as a biomarker for early pregnancy diagnosis is reported earlier in cattle [5,8], sheep [6] and goats [9,10].

The profiles of many hormones in circulation are comparable to the ones in milk. Therefore, concentration of a specific hormone in milk may be a good estimate of its level in the circulation [11]. Moreover, earlier studies in cattle indicate that the level of PAG in milk may reflect its blood concentrations [12,13]. In view of this, the use of milk PAG concentration for pregnancy diagnosis in goats may be preferred because collection of milk samples can be done by stress-free and non-invasive method, does not require special expertise and is easier to collect and store than blood. Milk is a complex and heterogeneous medium with fat globules, casein micelles, and cells in suspension in the liquid phase. Therefore, in order to avoid any possible interference due to these components during incubation steps in assay, skim milk is some time preferred for quantification of target molecules compared with the whole milk samples [14].

Thus, the objectives of the study reported herein were to i) analyse PAG profile in whole and skim milk samples using enzyme-linked immunosorbent assay (ELISA) for early pregnancy diagnosis in goats, ii) to compare results of the milk-based ELISA with those obtained from the TRUS, and iii) to identify the earliest and best suitable time point at which milk PAG could reliably use for pregnancy detection in goats.

## MATERIALS AND METHODS

### Experimental animals and their management

Fifteen healthy lactating multiparous goats (2 to 3 parity, weighing 35.4±1.7 kg) were assigned to the study and were maintained at the Institute flock of ICAR-Central Institute for Research on Goats, Makhdoom, Mathura, India. The animals were group housed and reared in semi-intensive system of management from 4 weeks before until end of the experiment with uniform nutritional conditions and free access to water. Goats were bred 10 to 12 h after onset of natural oestrus by superior breeding bucks. All the experimental procedures were carried out in accordance with the good veterinary practices and approved by the Animal Ethics Committee of the Institute.

### Milk sampling

Milk samplings were done on alternate day from d 10 to 30 and thereafter weekly till 51 d post-mating. Composite milk samples (≈ 35 mL, n = 210) were collected into 40 mL polypropylene milk-collection vials containing 50 μL of bronopol (2-bromo-2 nitropropane-1, 3-diol; 18% solution, Bronolab-W II, D&F Control Systems Inc., Dublin, CA, USA) as a preservative. Milk samples were immediately placed on ice and brought to the laboratory. Approximately half volume of the whole milk samples were skimmed by centrifugation at 5,000×*g*, 20 min at 4°C and skim milk (fat-free milk portion) was harvested between supernatant (fat layer) and the pellet (debris and cells). Both whole and skim milk samples were stored at −40°C until assayed by the ELISA.

### Ultrasonographic assessment

Ultrasonographic examination was performed in all the animals at d 40 post-mating by employing ultrasound scanner (Just Vision 200-Model SSA-320A, Diagnostic Ultrasound System, Toshiba, Japan) equipped with a real time convex array transrectal transducer (PVF-738F) of variable frequency (5 to 7 MHz), as described earlier [15]. Briefly, does were kept off feed 12 h prior to the scanning and all the examinations were carried out in standing posture after proper restraining of animals. After evacuating the rectum when necessary, the carboxymethyl cellulose gel lubricated transducer was introduced into the rectum and urinary bladder was located as non-echoic black area. The urinary bladder was kept as a land mark for identification of early pregnancy and/or any genital examination. During TRUS of pregnant uterus, anechoic embryonic vesicle (black) surrounding the echoic (white) elongated streak (foetus) extending more than half of foetal fluid in the centre was distinctly imaged. It was the first identifiable structure scanned during this period to confirm pregnancy.

### Establishment of milk PAG ELISA

A two-steps immunometric sandwich ELISA was established for detection of milk PAG using paired combination of caprine PAG specific rabbit anti-PAG polyclonal antisera generated against distinct purified PAG preparations i.e. PAG#707 (anti-caPAG_55+62 kDa_) and PAG#708 (anti-caPAG_55+59 kDa_) [16,17]. The characteristics of antigen preparations used to generate caPAG specific antisera (PAG#707 and PAG#708) are presented in Table 1. The antiserum PAG#707 was used for coating of microtiter plates and the biotin labelled purified immunoglobulin G (IgG) (PAG#708) was used as a conjugated detection antibody. For biotin labelling, the lyophilized antisera (PAG#708) was first reconstituted in 50 mM sodium bicarbonate buffer (pH 8.2) and the immunoglobulins were purified by using Protein-A based IgG purification kit (Himedia, MBPP001SP, Mumbai, India). After determination of total protein concentration by BCA method (Merck Millipore, 71285-3, Billerica, MA, USA), the purified immunoglobulins were labelled with biotin (Sigma, Mix-n-Stain Biotin antibody labelling kit; MXBIOS100, St. Louis, MO, USA), following manufacturer’s instructions.

For assay, high binding 96 well microtiter plates (EIA plate 9018; Corning Costar, Cambridge, MA, USA) were coated with rabbit anti-PAG polyclonal antibodies (PAG#707; 1:2,500) in coating buffer (phosphate-buffered saline [PBS]; pH 7.2) and allowed to incubate overnight at 4°C in moist condition. After blocking of free binding sites with 2.5% casein in 0.05 M NaCl, pH 7.3 (200 μL/well) at room temperature (RT) for 2 h, the plates were then washed 5 times with wash solution (250 μL/well for each wash; PBS containing 0.05% Tween 20) (Plate washer Hydroflex, Tecan, Seestrasse 103, 8708 Männedorf, Switzerland). After washings, the coated microtiter plates were used immediately for milk PAG assay.

First, 100 μL of whole milk or skim milk samples and assay negative control (pool whole or skim milk samples from non-pregnant goats; in six different predefined wells) were added in duplicate, and incubated for 90 min with shaking (250 rpm; Multiwall plate shaker, Tarson, Kolkata, India) at 37°C (Digital incubator T-701, Shivaki, New Delhi, India). After incubation, plates were washed three times with 250 μL wash solution and 100 μL biotin labelled anti-PAG antibody (AS#708; 1:5,000) dissolved in assay buffer (0.12 M NaCl, 0.02 M Na_2_HPO_4_, 0.01 M di sodium ethylenediaminetetraacetic acid, 0.005% chlorhexidine digluconate [20%], 0.002% phenol red, 0.1% gelatin, 0.05% Tween 20, and 0.02% ProClin 150) was pipetted into each well. After incubation for 1 h at 37°C (without shaking) and four times washing, 100 μL of detector solution (assay buffer with Avidin–Peroxidase, Sigma, A3151, USA) was pipetted and incubated for 1 h at 37°C (without shaking). After 5 washings, the substrate solution containing 0.05 M citric acid, 0.055 M Na_2_HPO_4_, 0.05 M H_2_O_2_, 0.02% ProClin 150, and 0.025% of TMB (12.5 mg 3,3′5,5′-Tetramethylbenyidine/mL dimethyl sulfoxide) was added and incubated for 30 min at RT in dark. The reaction was stopped with 50 μL of 1 M oxalic acid, and optical density (OD) was determined at 450 nm with a microtiter plate reader (Tecan Sun Rise with Magellan 4.0 Software, Austria).

### Result interpretation

Result interpretation was done as described earlier [5,17]. Briefly, OD values of each sample in duplicate and six ODs of negative control were measured at 450 nm and a reference wavelength of 630 (620 to 650) nm. Corrected OD values for all samples and controls were measured as the OD value at 450 nm minus the OD value at the reference wavelength (650 nm). A ‘signal minus noise (S-N)’ value was then determined for each samples by subtracting the mean of the corrected negative control OD from the mean corrected OD of the samples.

### Analytical validation

Analytical validation of the developed ELISA was achieved by determining reproducibility (coefficient of variation [CV] within a plate [intra-assay CV or repeatability] and between plates [inter-assay CV or precision] measured on different days, recovery and dilutional linearity. Furthermore, variables for assessment of predictive values of the assay were estimated.

#### Reproducibility (repeatability and precision)

Reproducibility was determined by repeated analysis of different milk samples measured in an assay or assays conducted on different days and expressed as the per cent intra-assay or inter-assay CV (% CV = [standard deviation / mean]×100), respectively.

For determination of repeatability (intra-assay precision), four milk samples with different PAG concentrations were tested at thirty-two different positions of two assay plates. Similarly, for the determination of inter-assay precision, sixteen samples were measured (in duplicate) in twelve ELISA plates on different days.

#### Recovery of the assay

The recovery was determined by the repeated analysis of three each of skim and whole milk samples with predetermined S-N values (high, mid and low). For this, six different combinations of these samples were prepared by the pool of two samples equally (50:50) at a time and measured in the assay. The percent recovery was determined by comparing the S-N values of assay with the predetermined values and calculated using the formula ([observed value − expected value]/expected value)×100.

#### Dilutional linearity

The dilutional linearity of the assay provides information about the precision of assay results for samples tested at different levels of dilution. The whole and skim milk samples were used undiluted as well as diluted with assay buffer in different concentrations (1:2, 1:3, 1:4, 1:5, and 1:6), and subjected to the ELISA tests. The dilutional linearity was calculated from the regression analysis and the calculation of CV of observed S-N values.

### Statistical analyses

For statistical analysis of time dependent changes in whole and skim milk PAG profile, the ‘linear mixed model procedure’ was used. Sample type (whole or skim milk) and pregnancy state (pregnant or non-pregnant) as fixed factor, sampling time (days post-breeding) as repeated effects, and their respective interactions were included into the model. The Bonferroni correction was used to estimate level of significance of pregnancy, time and their interaction for dependent variable (level of PAG in whole and skim milk). To account for multiple mean comparisons of milk PAG among the pregnant and non-pregnant groups, the Kolmogorov-Smirnov test was performed to determine whether the data was normally distributed. An independent sample t-test or corresponding non-parametric test (Mann–Whitney U test) was performed to assess the significant differences between overall mean at individual time point. Pearson’s correlation and simple regression analysis were done to establish agreement among whole milk and skim milk PAG levels measured by in-house developed and commercial ELISA. Differences between the groups were assessed by probability value (p) with the following levels of significance: p<0.05, p<0.01, p<0.001. All data are presented as a mean±standard error of the mean.

Pregnancy outcomes based on TRUS were considered as the reference test to which performance of the in-house milk PAG ELISA were compared by calculating the sensitivity, specificity, accuracy and the predictive values i.e. positive predictive value (PPV) and negative predictive value (NPV). A total 90 samples each of whole and skim (from d 26 to d 51) were included in this analysis. For this, data were arranged as follows: i) number of correct positive pregnancy diagnoses (PAG-ELISA test and TRUS examinations positive), ii) number of incorrect positive pregnancy diagnoses (PAG-ELISA test positive and TRUS examinations negative), iii) number of incorrect negative pregnancy diagnosis (PAG-ELISA test negative and TRUS examinations positive), and iv) number of correct negative pregnancy diagnosis (PAG-ELISA test negative and TRUS examinations negative).

From these data, i) the sensitivity = number of correct positive pregnancy diagnosis/number of all pregnant goats based on the TRUS×100 [a/(a+c)×100]; ii) The specificity = number of correct negative pregnancy diagnosis by the ELISA test/all non-pregnant goats based on the TRUS×100 [d/(d+b) ×100]; iii) the positive predictive (PPV) = number of correct positive pregnancy diagnosis/number of pregnant goats diagnosed by blood or milk test ×100 (a/[a+b]×100); iv) the NPV = number of correct negative pregnancy diagnosis/number of non-pregnant goats diagnosed by milk test ×100 (d/[c+d]×100); and v) the overall accuracy = number of correct positive pregnancy diagnosis + number of correct negative pregnancy diagnosis/total number of goats tested ×100 ([a+d]/ [a+b+c+d]×100). Also, 95%-confidence interval of each accuracy parameter of the diagnostic tests was determined [4].

SPSS-20 software was used to create receiver operating characteristic (ROC) curves where pregnancy was designated as the true positive. These curves (plots of sensitivity against [1-specificity]) were plotted to estimate area under curve (AUC) and to establish the optimal cut-off value of milk PAG based on the best combination of sensitivity and specificity [18]. The results of AUC were interpreted as follows: AUC = 0.5 (no discrimination), 0.7≤AUC<0.8 (acceptable discrimination), 0.8 AUC<0.9 (excellent discrimination), and AUC greater than 0.9 (outstanding discrimination). Estimates of the agreement between results of TRUS and milk PAG ELISA were determined by using Cohen’s kappa coefficient. A kappa value of 0.4 to 0.5, 0.5 to 0.6, and >0.6 indicates moderate, good and high degree of agreement, respectively [19]. Furthermore, a predictive model of group membership was built based on the observed PAG concentrations of each case to recognize the most appropriate timing of whole and skim milk sampling for predicting pregnancy in goats. This procedure generated a discriminant function using canonical discriminant function coefficients based on linear combinations of the predictor variables that provided the best discrimination between the sampling time points. The stepwise discriminant function analysis used in this study has been described elsewhere [20]. The discriminant function analyses produce a set of different values like eigenvalues, Wilks’ Lambda and canonical correlation values. These values provide an indication about the analytical importance of each predictor variable. The smaller values of Wilks’ lambda specify greater discriminatory ability of the function, whereas, a larger eigenvalue is associated with the strong function [21].

## RESULTS

**Figure 1:**
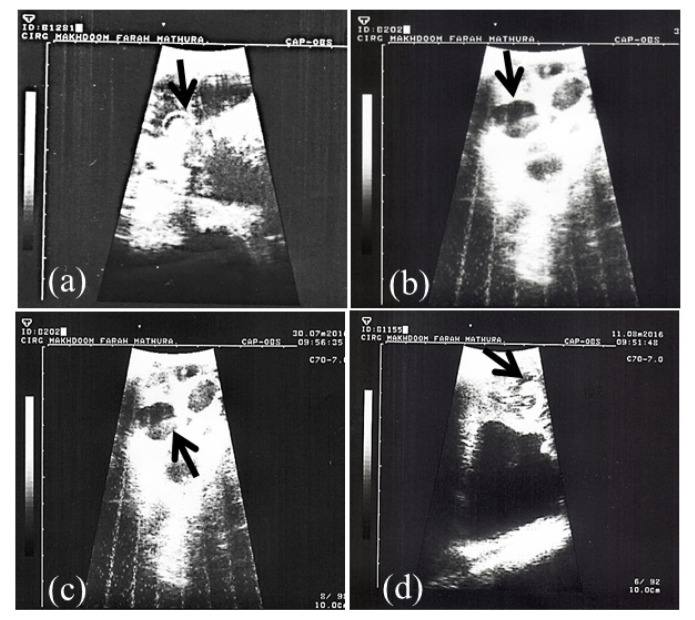
Transrectal ultrasonographic images (a–d) of pregnant uterus at d 40 of pregnancy in goats. Arrows in the ultrasound images indicate presence of echodense embryo with anechoic foetal fluid (b, c, d) and embryo encircled by distinct hyperechoic amniotic ring (a).

Pregnancy in goats was revealed by TRUS and PAG analysis in milk samples (whole and skim milk) through in-house ELISA methods. Representative ultrasonograms of uterus of goats depicting pregnancy are shown in Figure 1. Out of 15 goats, 12 were pregnant and 3 were non-pregnant.

### Validation of milk PAG ELISA

**Figure 2:**
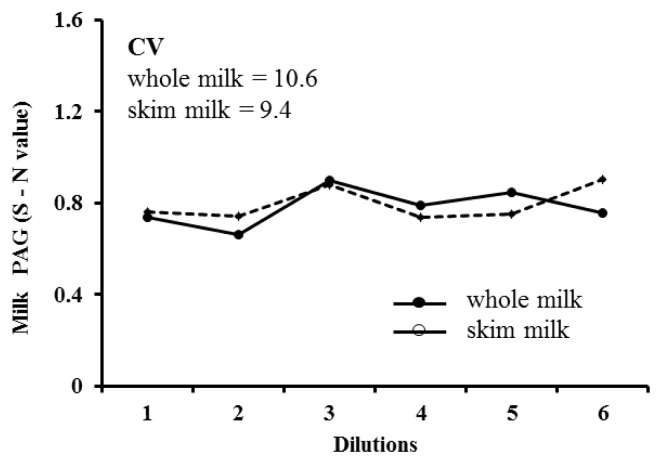
Representative serial dilution curve of whole milk (mean S-N = 0.78) and skim milk (mean S-N = 0.80) samples, showing linearity at different dilutions of a sample (undiluted, 1:2, 1:3, 1:4, 1:5, and 1:6) with assay buffer.

Intra- and inter-assay CVs of whole and skim milk were 6.9% and 8.5%, and 6.1% and 7.5%, respectively. The recovery ratio was ranged between 98.1% and 105.7% (mean, 102.1% ±2.2%), and 99.8% to 114.2% (mean, 108.7%±4.5%) for whole and skim milk, respectively. Regarding dilutional linearity, mean CV of diluted whole and skim milk samples with different PAG levels measured through ELISA were 10.6% and 9.4%, respectively. Representative serial dilution curve of a milk samples is presented in Figure 2. The CV of PAG concentrations measured in the serially diluted samples of whole milk (10.6) and skim milk (9.4) demonstrate good dilutional linearity in the assay (Figure 2). These values confirm that the additional dilution of milk samples using the assay buffer may not affect the accuracy of milk PAG assay.

The PPV, NPV, sensitivity, specificity and accuracy of PAG ELISA at different threshold values of PAG for whole and skim milk samples collected on and after d 26 of gestation are presented in Table 2. Based on the most appropriate combination of these validation parameters i.e. sensitivity, specificity, PPV, NPV and accuracy, 0.11 and 0.15 were identified as the cut-off values of S-N for whole and skim milk, respectively. For these cut-off values, the sensitivity, specificity and accuracy of the whole milk and skim assays were 82.5, 95.3, 94.5, and 82.4, 100, 95.4, respectively (Table 2). High values of AUC (0.904 and 0.922 for whole and skim milk, respectively), demonstrate outstanding discrimination ability of milk PAG assay. As measured by ELISA, strong positive correlation (0.893; p<0.001) and high kappa value (0.894; p<0.001) were observed between the PAG levels in whole and skim milk samples. These values reveal excellent agreement among the PAG levels in whole and skim milk of goat.

### Evaluation of the ROC curve and discriminant function analyses

**Figure 3:**
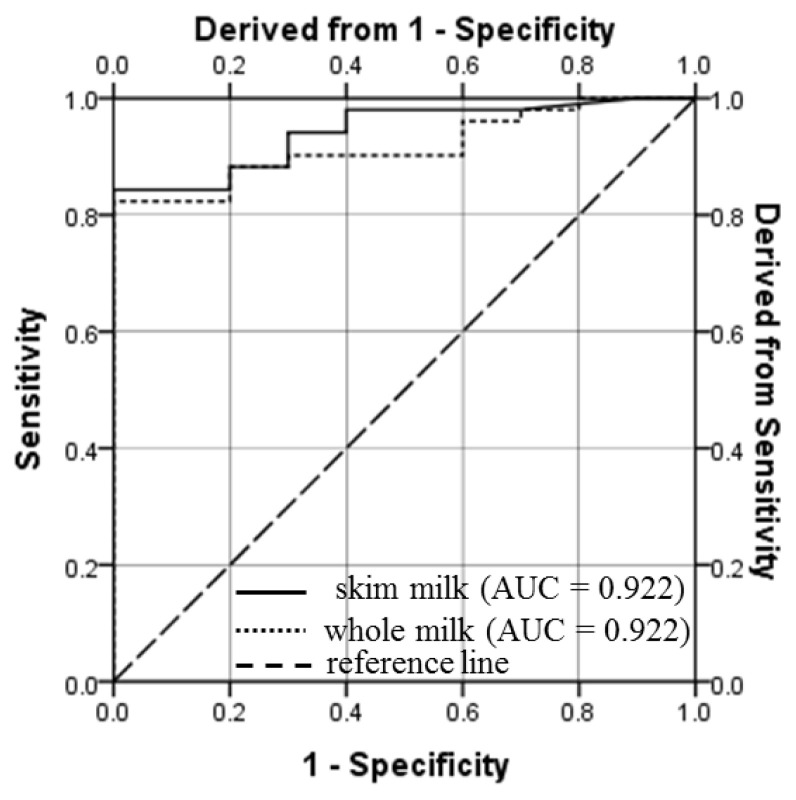
Receiver operating characteristic (ROC) curve for the enzyme-linked immunosorbent assay (ELISA) of pregnancy-associated glycoprotein (PAG) in milk samples of pregnant goats (from d 26 to 51 of gestation). The ROC curve is a graphical illustration created by the values of true positive rate and the false positive rate at different cut-off levels for the assay is applied. The assay with points on the reference line (diagonal dashed line) depicts a non-discriminatory test. The shifting of lines towards the upper left corner (a point where sensitivity and specificity are 100%), is an indication of the usefulness of the assay, because it represents a relatively high true positive rate and low false positive rate at a given cut-off value. The cut-off values of PAG in whole milk (S-N = 0.11) and skim milk (S-N = 0.15) assays resulted into 82.5% and 82.4% sensitivity, and 95.3% and 100% specificity, respectively. AUC, area under the curve.

One of the aims of our study was to determine S-N cut-off values of whole and skim milk for early pregnancy detection in goats. For this, ROC curve analysis was done and results are depicted in Figure 3 and Table 2. This analysis resulted in to an AUC of 0.904 (p<0.001) and 0.922 (p<0.001) for whole and skim milk assay, respectively (Table 2). The values of AUC indicate excellent discrimination ability of the PAG assay for whole and skim milk samples. When the analysis was conducted for each time-point, the AUC of the ROC curve remained ≤0.600 for PAG level in whole milk (0.333 to 0.583) and skim milk (0.296 to 0.600) samples from d 10 to d 26 of gestation. However, the acceptable and outstanding discrimination could be done by PAG level in whole and skim milk samples on and after d 28 (AUC>0.810) and d 37 (AUC >0.950), respectively.

### Milk PAG profile

**Figure 4:**
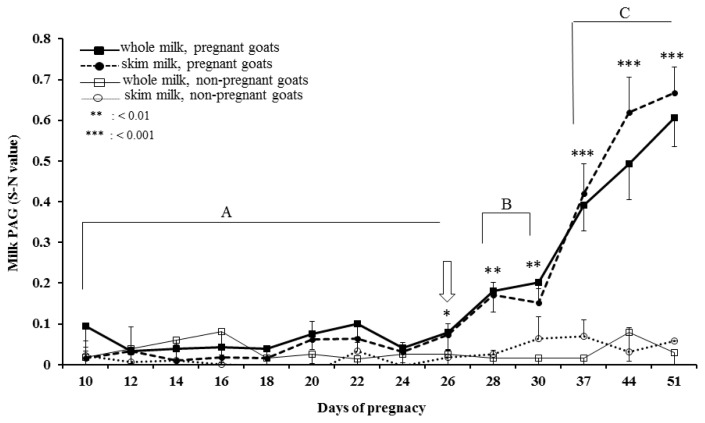
Dynamic profile of pregnancy-associated glycoprotein (PAG) in milk samples of pregnant and non-pregnant goats from d 10 to d 51 of gestation. Milk PAG enzyme-linked immunosorbent assay (ELISA) outcomes were calculated from the optical density (OD) of samples (S) minus the OD of negative control (N) at 450 nm and are presented as S-N value (Sample OD, negative control OD; both values corrected by subtraction of the reference wavelength OD [630 nm] of the negative control). Asterisk represents significant differences between pregnant and non-pregnant goats for whole and skim milk (* p<0.05, ** p<0.01, and *** p<0.001), and different letters (A, B, and C) represents significant (p<0.05) differences among different time points for milk samples of pregnant goats during early pregnancy.

**Figure 5:**
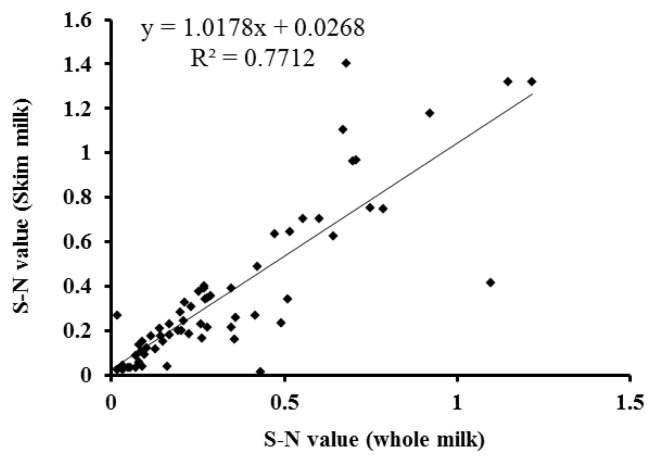
Relationship between the levels of pregnancy-associated glycoprotein (PAG) in whole and skim milk of pregnant goats measured by enzyme-linked immunosorbent assay (ELISA) from d 26 do 51 of gestation. The regression line is represented by the solid line in the graph.

Temporal profiles of PAG concentration in whole and skim milk of pregnant and non-pregnant goats during early pregnancy were analysed by ELISA and presented in Figure 4. The PAG profiles in whole and skim milk of pregnant goats are characterized by relatively unchanged up to about d 24 post-mating followed by gradual increase up to d 30 and thereafter rapid increase from d 30 to d 51 of gestation. Whereas, the concentration of PAG in milk samples of non-pregnant goats remain relatively at the same level below cut-off values throughout the study. The similar PAG profiles in whole and skim milk is demonstrated by high positive correlation (r = 0.891; p<0.001) and regression coefficient (R^2^ = 0.771; Figure 5). Moreover, the results of inter-test agreement for whole and skim milk pregnancy tests with TRUS are presented in Table 3.

Day 26 post-mating was identified as the first time point for significantly (p<0.05) higher PAG concentration in skim and whole milk of pregnant goats than the non-pregnant goats. The mixed model analysis along with pairwise comparison revealed significant difference between groups (pregnant and non-pregnant; p<0.001) and the sampling time points (p< 0.01) for both whole and skim milk, on and after d 26 post-mating. The results of discriminant function analyses for discrimination of whole and skim milk sampling days (d 30 and d 37) during pregnancy are presented in Table 4. Based on the values, skim milk samples at d 37 provide more accurate results for identification of pregnancy compared to the whole milk and the earlier time points. Furthermore, the results of discriminant function analysis revealed d 37 post-mating as the most suitable day of milk sampling, explaining 100% of variance and highest eigenvalue among all the time points, for prediction of pregnancy by milk PAG estimation (S-N value = 0.39±0.06 vs 0.42±0.07 for whole and skim milk, respectively) (Table 4).

## DISCUSSION

This study was designed to characterize PAG profile in whole and skim milk using an ELISA for detection of early pregnancy in goats. Further, the results were compared with the outcomes of the TRUS. Moreover, analyses were conducted to identify the earliest and best suitable time point at which milk PAG based pregnancy-detection assay could reliably identify pregnancy in goats. Regarding “spatial explanation” for the recognition of PAG molecules by the 2 different antibodies used, based on the results of the present study, we could not demonstrate if the epitopes are located in the C or in the N terminus of the PAGs. However, the PAGs used for the generation of antisera in rabbits were PAG_55+59 kDa_ and PAG_55+62 kDa_, so that, at least one major PAG of each preparation were different in molecular mass and N-terminal sequences. By this, we increased the probability of different PAGs (present in the goat milk) being detected by the present sandwich ELISA system.

The overall sensitivity of the ELISA for PAG analysis (whole milk, 82.5%; skim milk, 82.4%) is consistent with previously described sensitivities of milk PAG assays for sheep using different anti-PAG antisera (24% to 94%) [6], but lower than the values reported for dairy cattle using commercial assay i.e. 97.8% [13] and 99.7% [22]. The specificity of the PAG ELISA used in present investigation for whole milk (95.3%) and skim milk (100%) is in accordance with the earlier studies on sheep (66% to 100%) [6] and cattle (95.5% and 97.9%) [13,23], but higher than the specificity of milk PAG assay (80.8%) reported in another study on cattle [21].

High values of AUC for whole (0.904) and skim milk (0.922) along with high kappa value of whole (0.773; p<0.001) and skim milk (0.796; p<0.001) demonstrate high degree of agreement with results of TRUS and excellent discrimination ability of in-house milk PAG assay for detection of pregnancy in goats [19]. Overall, results of the present study suggest the use of in-house milk PAG ELISA on and after d 26 of gestation for pregnancy diagnosis in goats with high specificity and accuracy.

While conducting ELISA with milk samples interferences in antigen-antibody binding can be caused by milk fat and/or biomolecules attached with the fat globules. Removal of large part of fat content of milk samples by centrifugation may result into less complex matrix system of skim milk than of the raw whole milk [24]. Therefore, the effect of milk matrices on outcome of the PAG ELISA was tested by using whole and skim milk samples. Though the reproducibility (measured as intra- and inter-assays CVs) was slightly improved for skim milk compared with the whole milk samples, the recovery ratio was almost similar for all the matrices tested for whole and skim milk samples. The mean recoveries for whole (102.1% ±2.2%) and skim milk (108.7%±4.5%) samples confirm the accuracy of the ELISA for both the samples. Overall, the temporal profile of PAG concentrations in whole and skim milk of pregnant goats were almost similar (≥d 26), with strong positive correlation. This indicates that, the differences in the sample matrix due to skimming (removal of fat) did not affect the recovery ratios and concentration of PAG in milk samples. Whatever it may be, it is worth noting that fat content of milk did not preclude the binding to ELISA capture antibody or the binding of the detection antibody to the captured PAG molecule as it is reported for other hormones such as estradiol-17β and progesterone [25].

It has previously been reported that PAG after secretion are transferred to the milk [6,26]. Thus, in this study, we developed and validated a new ELISA system for estimation of PAG level in goat milk samples. Concentration of PAG in whole and skim milk samples of pregnant goats progressively increased from d 26 until d 51 whereas, in non-pregnant goats, PAG level remain below the threshold values from d 10 until end of the experiment. The pattern of progressive increase in concentration of milk PAG during early pregnancy is similar to the milk PAG profile observed in other farm animals *viz*. sheep [6,27] and cows [8]. Though, the transient decline in milk PAG concentration on d 49 and between d 32 to d 67 are described in sheep [6] and dairy cows [4], respectively. Such changes in temporal profile in milk PAG was not observed until d 51 of gestation in present investigation. This difference in the profile of PAG in milk may be due to the species-specific variation in expression and release of PAG into circulation, and matrix of the milk samples analysed.

In present experimental conditions, we observed d 26 of gestation is the first time point when PAG in whole and skim milk sample can be used for pregnancy diagnosis in goats. This is in accordance with the earlier study in which d 25 was identified as the earliest time point for pregnancy determination in sheep [6]. Whereas, in other studies on cattle d 30 was reported as the earliest time point at which pregnant cows were accurately identified by milk PAG ELISA [5,13]. In addition to the species specific difference in the PAG concentration in milk samples, this variation could be attributed to the difference in specificity of antisera used in the assay. The smaller values of Wilks’ lambda indicate better discrimination ability of the function used in the analysis [28]. Therefore, based on the results, d 37 post-mating was identified as the best suitable time point for detection of pregnancy using milk samples in goats (Table 4).

Although, the transportation mechanisms of PAG from maternal circulation to the milk is still unknown, Ali et al [29] assumed that the PAG as a water soluble protein may be able to cross the surface membrane lipid bi-layer and thus secrete out through the milk. The PAG in circulation is detectable around d 24 of gestation [30], therefore, measurable amount of PAG in milk is coincides with this physiological mechanism.

The correlation and kappa analysis were conducted to identify the relationship among PAG level of whole and skim milk samples measured with the assay. We observed strong positive correlation between PAG levels of whole and skim milk samples (r = 0.891; p<0.001). The results of correlation study along with the kappa statistic (kappa value = 0.894), indicate excellent agreement beyond chance between both the samples for PAG estimation. Overall, when compared with other available tools for pregnancy detection in goats, the PAG in milk could be a good alternative, being useful for determination of pregnancy on and after d 26 post-mating in goats.

In conclusion, the early pregnancy detection in goats based on PAG concentration in whole and skim milk was accurate between days 26 and 51 post-breeding. Day 37 post-mating was identified as the best suitable time point for pregnancy diagnosis using milk samples in goats. Strong positive correlation coupled with high kappa value and similar temporal profiles indicate suitability of both whole and skim milk samples for PAG estimation and early pregnancy diagnosis in goats. Further investigation into the association of milk PAG with early embryonic mortality may provide valuable tool to study the functioning of foeto-placental unit in goats.

## Figures and Tables

**Table 1:** Antigen preparations used to generate caprine pregnancy-associated glycoprotein (caPAG) specific antisera used in the milk enzyme-linked immunosorbent assay

Antisera	PAG	N-terminal micro-sequence	Accession No.
707	caPAG_55kDa_	ISSPVSXLTIHPLRNIMDMLYVGXITI	P80935
	caPAG_62kDa_	RDSXVTIVPLRNMRDIVYVGXITIGTP	P80933
708	caPAG_55kDa_	ISSPVSXLTIHPLRNIMDMLYVGXITI	P80935
	caPAG_59kDa_	RGSXLTTLPLRNIMDMLHMGXITIGTP	P80934

**Table 2:** Sensitivity, specificity, PPV1), NPV2), and accuracy3) of the in-house developed milk PAG ELISA test for pregnancy diagnosis based on the various threshold values of whole and skim milk PAG level (S-N) assessed during early pregnancy in goats

Days of pregnancy	PAG-threshold values	Se (%)	Sp (%)	PPV (%)	NPV (%)	Accuracy (%)	AUC (p-value)	95% CI	

								Lower bound	Upper bound
Whole milk (≥26 days)	0.081	87.3	69.2	94.0	85.7	92.1	0.904	0.831	0.977
	0.11	82.5	95.3	100	84.6	94.5	(p<0.001)		
	0.18	68.4	92.3	92.2	51.4	75.6			
Skim milk (≥26 days)	0.10	88.2	83.3	92.3	79.2	92.9	0.922	0.856	0.987
	0.15	82.4	100	100	83.3	95.4	(p<0.001)		
	0.17	78.4	100	87.5	70.4	89.3			

PPV, positive predictive value; NPV, negative predictive value; PAG, pregnancy-associated glycoprotein; ELISA, enzyme-linked immunosorbent assay; Se, sensitivity; Sp, specificity; AUC, area under the curve; CI, confidence interval.

1) Proportion of goats diagnosed pregnant using PAG that truly were pregnant.

2) Proportion of goats diagnosed as not pregnant using PAG that truly were not pregnant.

3) Proportion of pregnancy status, pregnant and not pregnant, that was correctly classified by the assay.

**Table 3:** Cohen’s kappa1) statistic for milk pregnancy tests and TRUS measuring inter-test agreement at ≥26 d of pregnancy for PAG and 40 d after mating

Item	TRUS	Whole milk PAG assay	Skim milk PAG assay
Skim milk PAG assay	0.796	0.894	1.000
Whole milk PAG assay	0.773	1.000	-
TRUS	1.000	-	-

TRUS, transrectal ultrasonography; PAG, pregnancy-associated glycoprotein.

1) Cohen’s kappa = test of agreement between the outcome of assays.

**Table 4:** Discriminant function analyses results for discrimination of day of whole and skim milk sampling during pregnancy

Cononial discriminant function coefficients	d 30 of gestation		d 37 of gestation	
	
	Whole milk	Skim milk	Whole milk	Skim milk
Wilks’ Lamda value	0.792	0.865	0.619	0.589
Unstandardized coefficients	−1.062	−1.269	−1.541	−1.634
Chi-square	2.921	1.520	5.993	5.034
Function at group centroids				
Pregnancy				
Pregnant	6.455	9.627	4.864	0.441
Non-pregnant	−1.062	−1.269	−1.541	−1.322
Eigenvalue	0.263	0.156	0.615	0.699
% of variance	100	100	100	100
Canonical correlation	0.457	0.478	0.617	0.641
